# Tailoring Architecture of Carbon Aerogel via Self-Assembly Template for Balanced Mechanical and Thermal Insulation Performance

**DOI:** 10.3390/nano15241874

**Published:** 2025-12-13

**Authors:** Lei Yang, Xianxin Shao, Lin Lu, Xiaoyan Chen, Yiming Yang, Hao Li, Yiqiang Hong, Yingjie Qiao

**Affiliations:** 1 College of Material Science and Chemical Engineering, Harbin Engineering University, Harbin 150001, China; 2 Beijing System Design Institute of Electro-Mechanic Engineering, Beijing 100854, China; 3 Key Laboratory of Science and Technology on High-Tech Polymer Materials, Institute of Chemistry, Chinese Academy of Sciences, Beijing 100190, China; shaoxianxin@iccas.ac.cn (X.S.); lihao306@iccas.ac.cn (H.L.)

**Keywords:** carbon aerogel, mechanical properties, self assembly, nano crystalline, closed pores

## Abstract

Carbon aerogels (CAs) had been well applied in extreme condition thermal insulation, but achieving a balance between mechanical robustness and thermal insulation remains challenging. We present a novel strategy to fabricate carbon aerogels with tunable mechanical properties and thermal insulation properties by tailoring their skeleton architecture via molecular assembly. Carbon precursor aerogel with thick neck particle packing structure was obtained by strong hydrogen-bonding-induced self-assembly between polyurethane-urea oligomer (PUU) and phenolic resin (PF), and carbon aerogel retained robust interparticle connections after pyrolysis, resulting in excellent mechanical properties. The presence of PUU leads to denser packing of resin molecules, promotes graphitization of the carbon and formation of nanocrystalline structures at 1400 °C, resulting in optimized compression modulus and strength. The closed pore structure of carbon skeleton was further studied by Small-Angle X-ray Scattering (SAXS), while moderate pore width (0.4–0.6 nm) optimizes the balance between strength (110 MPa) and thermal conductivity (0.30 W/(m·K)). This work demonstrates that molecular-level assembly combined with pyrolysis control enables precise tuning of carbon aerogel structures and properties, providing new insights for high-temperature thermal insulation applications.

## 1. Introduction

Carbon aerogels, as a class of nanostructured porous carbon materials with ultralow density, high porosity, and continuous three-dimensional networks, have garnered immense attention in fields spanning aerospace thermal insulation, energy storage, environmental remediation, and advanced catalysis [1,2]. Their unique combination of structural features, including nanoscale building blocks, interconnected porous architectures, and tunable carbon microstructures, endow them with excellent properties that bridge the gap between traditional porous materials and cutting-edge nanodevices [3,4,5,6]. However, owing to high porous carbon skeleton network and weak interparticle joints, the mechanical properties of CA still need to improve as load-bearing materials [7,8]. Also, the practical application of carbon aerogels is often hindered by the lack of a clear understanding of the relationship between their microstructures and macroscopic properties, particularly in balancing critical performances between mechanical robustness and thermal insulation, which are crucial for extreme-environment applications such as high-temperature thermal protection systems in aerospace [9,10,11,12].

To overcome the intrinsic fragility of carbon aerogels, multiple reinforcement strategies have been pursued in recent years [13,14]. One approach is structural tailoring of the carbon structure at the nano and micro levels to produce a more robust skeleton. Direct skeleton modulation via templating has enabled carbon aerogels with enhanced structural connectivity and minimal drying shrinkage [15]. Schwan et al. reported resorcinol–formaldehyde aerogels prepared by tuning sol–gel parameters of reactant ratio, catalyst, and pH, achieving order-of-magnitude density reduction and elastically recoverable strains >40%, thereby converting classically brittle RF aerogels into flexible monoliths that can be carbonized into CAs with preserved integrity [16]. Morales-Torres et al. reported resorcinol–formaldehyde-derived carbon aerogels in which using different alkali carbonates to modulate gelation process to form cluster structure, resulted in increased Young’s modulus after carbonization, evidencing catalytic control of network topology [17]. Li et al. reported aerogel-like C/C monoliths fabricated by high-pressure-assisted polymerization promoting formation of intact robust network to resist shrink during ambient-pressure drying, the carbon skeleton showed enhanced connectivity and large, crack-free, load-bearing C/C can provide thermal insulation up to 1800 °C [18]. Wang et al. reported a vacuum-impregnation-assisted nano-repairing (VINR) route to enhance CA composites, repairing of microcracks on carbon-skeleton-endowed CA composites with ultrahigh strength, superior thermal insulation, and EMI shielding [19]. Overall, such structural tailoring strategies, including sol–gel chemistry, templating, and hierarchical design, directly reinforce the CA’s internal network and have substantially improved compressive strength and dimensional stability of carbon aerogels [18,20,21,22,23].

Similarly, embedding nano or microscale reinforcements into the carbon aerogel skeleton can further enhance mechanical properties. Incorporation of organic fiber into carbon aerogel precursor followed by co-pyrolysis can reduce cracking or interface detach caused by incompatible shrinkage. Feng et al. reported CA composites from polyacrylonitrile fiber-reinforced RF aerogels; a shrinkage-mismatch strategy was deployed to obtain crack-free monoliths with low thermal conductivity of 0.073 W m^−1^ K^−1^ and improved mechanical integrity [6]. Ma et al. reported fiber-reinforced “aerogel-like” carbon matrix composites prepared from phenolic resin fiber felt to realize co-pyrolysis with matrix, then used microstructure tailoring via higher carbonization temperatures (≤1200 °C) to achieve compressive strength 45.8–96.9 MPa (ρ ≈ 0.58–0.64 g cm^−3^) [24]. Li et al. reported an exceptionally strong and damage-tolerant carbon–aerogel composite prepared by densifying and toughening the aerogel network around fibers; then used a multiscale network design to achieve compressive strength of 328 MPa and fracture energy of 42 MJ m^−3^, and thermal stability/insulation at high temperature [25]. Zhang et al. reported a carbon-fiber-reinforced carbon aerogel prepared by impregnating polyacrylonitrile fiber felts with a resorcinol resin sol with presence of salt as nanopore generator; this design endowed CA composites with enhanced compressive strength as well as extremely low thermal conductivity of 0.69 W m^−1^ K^−1^, even at 1800 °C [22]. Graphene-based toughening has also proven effective: Gao et al. designed a binary carbon aerogel consisting of intertwined graphene sheets and carbon nanotubes, which can sustain up to 200% tensile strain and millions of compression cycles without failure [26]. Zhou et al. reported expandable-graphite-optimized CA composites where graphite serves as an interfacial binder within the multiscale carbon network; the resulting composites exhibited compressive strength of 226.9 MPa and modulus of 1935 MPa while maintaining high-temperature insulation [27]. By combining these reinforcements method including structural tailoring, fiber integration, and nanoscale toughening, recent advances have produced carbon aerogel composites that are mechanically robust yet retain the hallmark low weight and thermal insulation of CAs [28,29,30,31]. The enhanced structural integrity and thermal performance of these carbon aerogels significantly broaden their application potential, particularly in advanced thermal protection systems, high-temperature industrial insulation, and other extreme environments where conventional aerogels typically suffer from mechanical fragility and thermal instability.

Despite notable progress in tailoring the structure and properties of carbon aerogels, most existing approaches rely on multi-step and intricate processing routes to modify the carbon structure. Moreover, a comprehensive understanding of how particle packing, closed pore architecture, and graphitization collectively influence mechanical integrity, and thermal insulation remains limited. Bridging this knowledge gap is essential for transitioning carbon aerogels from laboratory to practical applications, particularly in demanding environments where both structural resilience and thermal control are critically required.

In this work, we present a novel strategy to fabricate carbon aerogels with tunable mechanical properties and thermal insulation properties by tailoring their backbone architecture. Carbon precursor aerogel (Pre CA) with thick neck particle packing structure was obtained by strong hydrogen-bonding-induced self-assembly between polyurethane-urea oligomer (PUU) and phenolic resin (PF), and carbon aerogel (CA) retained robust interparticle connections after pyrolysis, resulting in excellent mechanical properties. The presence of PUU leads to denser packing of resin molecules, promotes graphitization of the carbon and formation of nanocrystalline structures at 1400 °C. PF-PUU CA showed enhanced mechanical strength and toughness. The closed pore structure of carbon was further studied by SAXS, while moderate pore width (0.4–0.6 nm) optimizes the balance between strength (110 MPa) and thermal conductivity (0.30 W/(m·K)). By dissecting the underlying mechanisms through which these structural features regulate macroscopic behaviors, this study establishes foundation for rational design and performance optimization of CAs, thereby promoting their broader implementation in high-performance thermal and structural applications.

## 2. Materials and Methods

### 2.1. Materials

Phenolic resin (PF) was purchased from Shandong Shengquan Group Co., Ltd. (Jinan, China). Hexamethylene tetramine was obtained from Sinopharm Chemical Reagent (Shanghai, China). Toluene-2,4-diisocyanate was obtained from Wanhua Chemical Group Co., Ltd. (Yantai, China). Poly(ethylene glycol) (PEO) with a molecular weight of 1000 g/mol was obtained from Shanghai Aladdin (Shanghai, China). 3-aminophenol was obtained from Innochem (Beijing, China). 2-propanol and ethanol were purchased from Beijing Chemical Works (Beijing, China). All materials were used as received without further purification.

### 2.2. Synthesis of PUU and PF

PEO (mol) was added into the three-necked flask with a mechanical stirrer and vacuumed at 110 °C for 2 h to remove traces of water. Then, 2,4-TDI (mol) with THF was added dropwise to the reaction flask and reacted at 75 °C for 2 h under N_2_ and continuous stirring to prepare isocyanate prepolymer. The system was cooled down to 50 °C followed by addition of 3-Aminophenol/THF solution into flask for 1.5 h at 50 °C. Strictly maintain the molar ratio of PEO/TDI/3-aminophenol at 1:2:2 to guarantee complete end-capping of the reaction. Afterwards, the solvent was removed by rotary evaporation. The obtained product was referred to as PUU. PF was synthesized via a base-catalyzed condensation of phenol and formaldehyde at a molar ratio of 1:2 (F:P). Phenol was first dissolved in deionized water and heated to 60 °C under continuous stirring. Formaldehyde solution (37 wt%) was then added dropwise, followed by the addition of Sodium Hydroxide (5% of phenol molar ratio) as catalyst °C. The reaction mixture was maintained at 80 °C for 2 h to promote hydroxymethylation of phenol. Subsequently, the temperature was raised to 95 °C and held for another 2 h to facilitate condensation and gradual removal of water generated during the polycondensation reaction. The resulting reddish-brown PF sol was cooled to room temperature and stored for further use.

### 2.3. Preparation of CA

Precursor aerogels were produced by the sol–gel process, followed by ambient pressure drying. PF and PUU with different weight ratio were added into 2-propanol with magnetic stirring for 60 min to form a uniform solution, and then sulfuric acid (2 wt% of solution) was added. The whole solution was stirred for 20 min. Subsequently, solution was transferred into an oven and heated to 90 °C for 24 h. The samples were dried at room temperature for 12 h and then dried at 70 °C for 24 h to yield precursor carbon aerogel (Pre CA). After that, Pre CA was carbonized in N_2_ at a flow rate of 300 mL/min, with heating rate of 2 °C/min and final temperature was set as 800, 1000, 1200, 1400, and 1600 °C, respectively. The corresponding CA was named as CA800, CA1000, CA1200, CA1400, and CA1600, respectively.

### 2.4. Characterization

The surface morphology of the samples was examined using a scanning electron microscopy (FE-SEM, Hitachi SU-8020, Tokyo, Japan), the sample was sput-coated before characterization. The detailed microstructure was further analyzed by high-resolution transmission electron microscopy (HRTEM, JEOL JEM-F200, Tokyo, Japan) combined with selected area electron diffraction (SAED). The crystalline structure was identified by X-ray diffraction (XRD, Rigaku SmartLab, Tokyo, Japan) operated at a scan rate of 6° min^−1^. Raman spectra were recorded on a LabRAM HR Evolution spectrometer (Longjumeau, France) with a 532 nm excitation laser (5 mW, sample surface power) at an exposure time of 30 s per scan and two accumulations. The skeleton density and pore-size distribution were evaluated using a Mercury Intrusion analyzer (Micromeritics ASAP 3030, Norcross, USA). The closed pore structure was characterized by Small-Angle X-Ray Scattering (SAXS, Xeuss 2.0, Grenoble, France). The thermal conductivity at room temperature was measured using a Hot Disk thermal constant analyzer (TPS 2500S, Gothenburg, Sweden) based on the transient plane source (TPS) method. The compressive mechanical properties were measured using a universal testing machine (Instron 5567, Boston, USA) at a constant loading rate of 1 mm·s^−1^. Cylindrical specimens with a diameter of 17 mm and a height of 17 mm were used for all compression tests to ensure reproducibility. The stress–strain curves were recorded automatically, from which the compressive strength and modulus were determined.

## 3. Results and Discussion

### 3.1. Molecular Assembly and Polymerization Mechanisms

**Figure 1:**
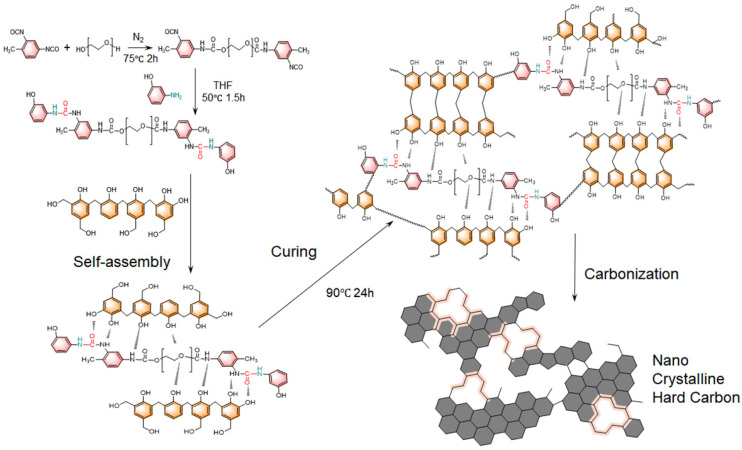
Schematic illustration of chemical reaction during CA preparation.

**Figure 2:**
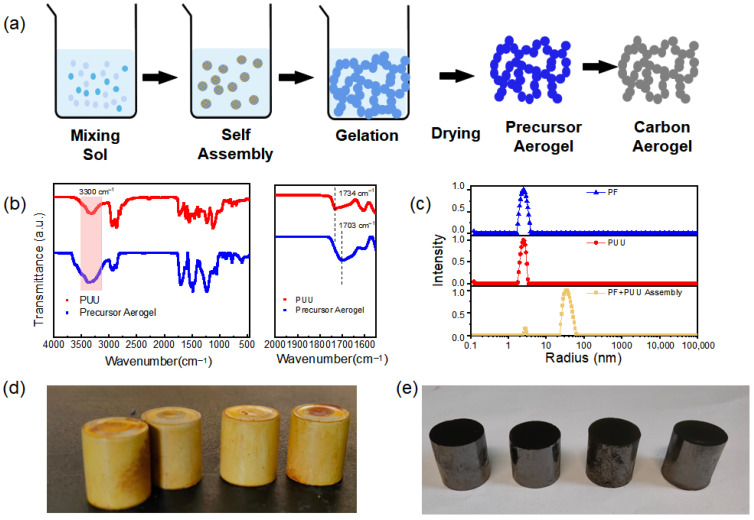
(**a**) Schematic illustration of CA preparation, (**b**) FTIR of PUU and Pre CA, (**c**) dynamic light scattering analysis of PF, PUU and self-assembled structure of PF-PUU in iso-propanol, picture of (**d**) Pre CA and (**e**) CA after carbonization at 800 °C.

The preparation of CA was illustrated in Figure 1. First, a polyurethane-urea oligomer (PUU) was synthesized by modifying polyethylene glycol (PEG) with toluene diisocyanate (TDI) and subsequently end-capped with 3-aminophenol. In this step, the hydroxyl-terminated PEG reacts with TDI to form isocyanate-terminated PEG chains, which are then capped by reacting with 3-aminophenol to prevent undesired polymerization from isocyanate. This creates PEG-based molecules bearing urethane (from the PEG–TDI reaction) and urea (from the TDI–3-aminophenol reaction) linkages at both ends. These terminal urea and urethane groups can form strong intra and intermolecular hydrogen bonds, which serve as the primary driving force for the self-assembly of PUU chains in the subsequent Pre CA formation. Next, a carbon precursor alcogel is formed by the co-polymerization of the phenol-formaldehyde (PF) resin with the PUU in solution. The polymerization of the PF resin proceeds mainly via condensation reactions between phenolic rings and hydroxymethyl groups (Figure S1). Hydroxymethyl-functionalized aromatic rings react with other active aromatic sites with elimination of water, forming methylene bridges that crosslink the growing network. Through this polycondensation, the PUU’s phenolic end-groups become chemically integrated into the PF resin matrix, creating an interpenetrating network. Throughout this process, the hydrogen-bond-driven self-assembly of PUU components helps organize the structure on a molecular level, while the PF crosslinking builds a rigid scaffold around and through the self-assembled PF-PUU. As the PF-PUU polymerization progresses, a reaction-induced phase separation occurs (Figure 2a). The initially homogeneous solution segregates into a solid polymer-rich phase and a solvent-rich phase. The polymer-rich phase forms an interconnected skeleton of colloidal particle, which acts as the skeleton of the Pre CA gel. This phase separation yields a continuous porous network formed by the agglomeration of the polymer particles, while the separated solvent regions will become pores later. After the polymerization, the wet gel is subjected to ambient-pressure drying, which removes the solvent and preserves the porous structure. Finally, the dried Pre CA is carbonized to yield CA. During carbonization, the organic network is converted into nanocrystalline hard carbon framework, largely retaining the original gel’s structure. The final CA thus consists of a highly porous network of carbon nanoparticles, derived from the PF-PUU polymer network, where the hydrogen-bond-directed self-assembly and in situ PF condensation have produced a uniform, nanoscale structure. This structure combines the benefits of both components, the robust carbon framework and the hierarchical ordering imparted by the self-assembled hydrogen-bonded PUU domains.

As illustrated in Figure 2a, the preparation route for the carbon aerogel is both facile and energy efficient. The gelation and curing processes proceed at a relatively low temperature of 90 °C, and the entire synthesis avoids time-consuming solvent exchange or supercritical drying. Notably, the Pre CA exhibited negligible shrinkage during ambient pressure drying, highlighting the mechanical robustness and dimensional stability of the pre-carbonized framework. To elucidate the self-assembly behavior, dynamic light scattering (DLS) and Fourier-transform infrared spectroscopy (FTIR) analyses were conducted. FTIR spectra (Figure 2b) confirm the formation of hydrogen bonding as the characteristic C=O stretching vibration of the PUU component shifts from 1703 cm^−1^ to 1734 cm^−1^ upon mixing with PF, indicating the participation of the carbonyl group in hydrogen bonding interactions. Self-assembly was further probed by DLS. As shown in Figure 2c, individual PF and PUU sol solutions displayed hydrodynamic radii (Rh) of approximately 3.0 nm and 2.4 nm, respectively. Upon mixing, the PF and PUU sols in ethanol, a new and significantly larger diffusion mode emerged, corresponding to an Rh of 34 nm, indicating the formation of supramolecular aggregates. This result provides direct evidence for hydrogen-bonding-driven assembly between PF and PUU components. The spontaneous assembly is attributed to the strong hydrogen bond donors present in the PUU chains (urea and urethane groups) and the multiple hydroxyl and phenolic groups in PF, which act as complementary acceptors and donors. These findings collectively confirm that the intermolecular hydrogen bonding between PF and PUU is the dominant driving force for the formation of a well-organized precursor network. The density of obtained Pre CAs is 0.35–0.38 g/cm^3^, and the corresponding CAs showed density between 0.56 and 0.63 g/cm^3^, varied with different carbonization temperature. The picture of CA was shown in Figure 2e, the carbon aerogel remained a cylinder shape without any crack. This approach offers a simple, scalable, and energy-efficient route for fabricating high-performance carbon aerogels without the need for complex drying processes or structural templates.

### 3.2. Morphology Analysis of Carbon Aerogels

**Figure 3:**
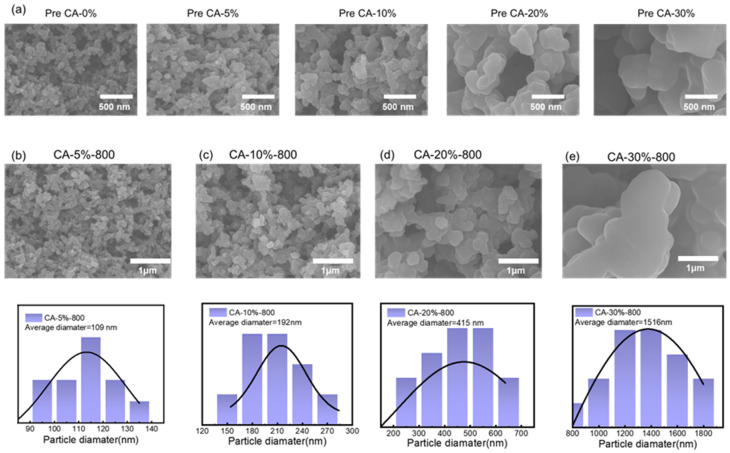
(**a**) Structure evolution of Pre CA with different PUU content, morphology, and particle diameter distribution of (**b**) CA-5%-800, (**c**) CA-10%-800, (**d**) CA-20%-800, (**e**) CA-30%-800.

The morphology evolution of Pre CA and their corresponding CA as a function of PUU content is presented in Figure 3a,b. With increasing PUU loading (5%, 10%, 20%, and 30%), the precursor gel network exhibited dramatic changes in particle size and interconnection patterns. Specifically, CAs showed similar structure evolution as they inherit similar particles’ packing structure from Pre CAs. SEM images and the corresponding size distribution histograms show that the average particle size of CAs increased substantially from 109 nm (5% PUU) to 192 nm (10%), 415 nm (20%), and up to 1516 nm (30%). This clear size amplification suggests that the amount of PUU significantly influences the colloidal aggregation dynamics during gelation, due to its role in mediating hydrogen-bonding-driven self-assembly and modulating the phase separation behavior of the sol system.

As shown in Figure 3b, the skeleton structure became bulky and continuous with PUU addition. At low PUU content (5%), the particles are small and loosely packed, forming a “pearl-necklace”-type framework with weak interparticle contacts. As PUU content rises, the interparticle “necks” become thicker and more fused, giving rise to a dense, integrated packing structure with stronger interparticle bridging. At 30% PUU, the particles coalesce into smooth, large granular domains with significantly reduced surface roughness and fewer visible pore boundaries, indicative of a transition from nanoscale to microscale structural units. This morphological shift reflects an enhanced phase separation degree at higher PUU concentrations, resulting in less nano level porosity. The corresponding carbon aerogels retained these microstructural differences after pyrolysis, further confirming the structural inheritance from precursor to carbonized state. These observations collectively demonstrate that PUU content serves as a molecular-level regulator, enabling precise control over particle size, skeleton connectivity, and packing morphology within the aerogel network. The ability to tune the skeletal morphology through PUU content, without the need for external templating or complex drying techniques, demonstrates the effectiveness of this molecular assembly strategy in directing nanoscale structure and ensuring its mechanical performance.

### 3.3. Influence of Pyrolysis Temperature on Graphitization and Mechanical Properties

**Figure 4:**
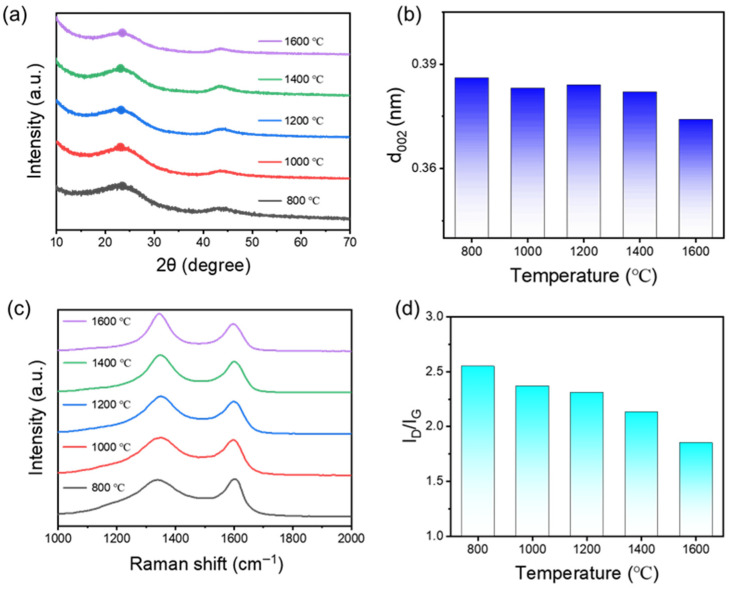
(**a**) XRD pattern and (**b**) calculated d_002_ of CA prepared at different temperature, (**c**) Raman spectra and (**d**) calculated I_D_/I_G_ of CA prepared at different temperature.

Pyrolysis temperature has a significant impact on the structural ordering and graphitization degree of CAs. As shown in Figure 4, both Raman spectroscopy and X-ray diffraction (XRD) analyses reveal progressive structural evolution with increasing carbonization temperature from 800 °C to 1600 °C. XRD patterns (Figure 4a) display the characteristic (002) diffraction peak of turbostratic carbon, and the corresponding interlayer spacing d_002_ gradually decreases with increasing temperature. Specifically, d_002_ values change from 0.384 nm at 1200 °C to 0.374 nm at 1600 °C, approaching but still above the ideal graphite value of 0.335–0.340 nm (Figure 4b). At 800 °C, the (002) peak remains broad and centered around 22.48°, indicative of a poorly ordered hard carbon structure. The relatively small shift in d-spacing suggests that although some rearrangement and partial stacking of graphene layers occur at higher temperatures, the overall graphitic ordering remains limited, consistent with the typical behavior of carbon derived from phenolic resin precursors.

Raman spectra (Figure 4c) show a more pronounced carbon crystalline evolution. The intensity ratio of the disorder-induced D-band (~1350 cm^−1^) to the graphitic G-band (~1580 cm^−1^), denoted as I_D_/I_G_, decreases significantly from 2.55 at 800 °C to 1.85 at 1600 °C (Figure 4d). This decline indicates a substantial reduction in defect density and a corresponding increase in graphitic domain size. While the XRD data suggests moderate ordering at the nanocrystalline scale, the Raman data reflect local bonding environment improvements and a reduction in amorphous carbon content with increasing pyrolysis temperature. Taken together, these results demonstrate that although phenolic resin-based carbon aerogels typically form hard carbon structures with limited graphitic stacking, thermal treatment above 1200 °C can significantly improve the in-plane ordering, as reflected in Raman results. The incorporation of PUU not only modulates the precursor’s microstructure but also enhances the degree of structural ordering upon carbonization, offering a feasible route to engineer nanocrystalline carbon frameworks with improved mechanical performance.

**Figure 5:**
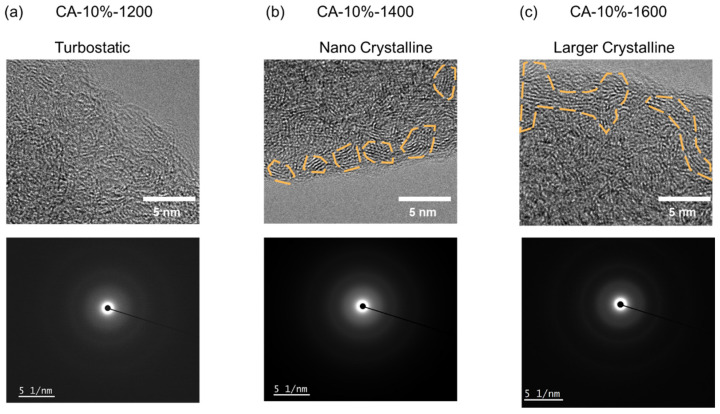
HRTEM analysis and SAED of carbon nanostructure of (**a**) CA-10%-1200, (**b**) CA-10%-1400, (**c**) CA-10%-1600.

To further investigate the structural evolution of the carbon aerogels during pyrolysis, high-resolution transmission electron microscopy (HRTEM) was conducted on samples treated at 1200 °C, 1400 °C, and 1600 °C (Figure 5). The results reveal transformation from turbostatic carbon to nanocrystalline domains, consistent with the Raman and XRD observations. At 1200 °C, the carbon structure remains largely disordered, with randomly oriented and curved graphene layers visible throughout the matrix. No significant lattice alignment or long-range order is observed, confirming that the material still predominantly retains turbostatic hard carbon features. This supports the Raman data showing a relatively high I_D_/I_G_ ratio (2.31) and the broad (002) peak in XRD centered around d_002_ = 0.384 nm. In contrast, at 1400 °C, distinct graphitic nanocrystals begin to emerge. These domains, typically ranging from 2 to 3 nm in lateral size, are highlighted in the image and exhibit partially aligned edges with local stacking. This is related to the particle-packing structure formed by PUU and PF through thick interparticle necks, leading to denser molecular packing and the creation of robust, thick interparticle necks. This reduces structural defects and enhances load-bearing continuity. Such nanocrystalline features represent emerging ordering within the carbon matrix, as graphene sheets begin to coalesce into localized crystalline regions. The corresponding Raman I_D_/I_G_ = 2.13 further confirms the partial graphitization at this stage. Upon increasing the carbonization temperature to 1600 °C, the carbon aerogel exhibits a significantly enhanced degree of ordering. HRTEM images reveal extended graphitic region, spanning over 10 nm in length, with more continuous stacking and reduced curvature. These interconnected nanocrystalline domains indicate the development of a quasi-graphitic framework, approaching the onset of long-range order. The accompanying reduction in I_D_/I_G_ and narrowing of the XRD (002) peak further corroborate this structural transformation. Selected area electron diffraction (SAED) patterns also support this trend. While the 1200 °C sample displays diffuse rings characteristic of amorphous carbon, the 1400 °C and especially 1600 °C samples exhibit sharper diffraction rings, indicating improved crystallinity and increased domain size. These findings highlight that although phenolic-resin-derived aerogels tend to form hard carbon, the molecularly guided self-assembled network and elevated local density introduced by the PUU component facilitate nanocrystal formation at relatively lower temperatures of 1400 °C. The ability to tune and stabilize these structural units offers a pathway to engineer mechanically robust, thermally resilient carbon aerogels with hybrid amorphous/graphitic characteristics.

**Figure 6:**
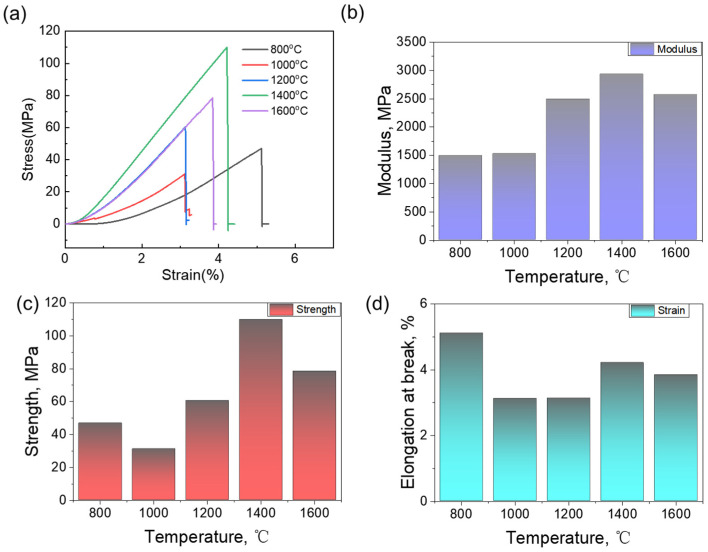
(**a**) Compression stress–strain curves of CAs, (**b**) Young’s modulus of CAs at different temperature, (**c**) strength of CAs at different temperature and (**d**) elongation at break of CAs at different temperature.

The compressive mechanical properties of the carbon aerogels exhibit a strong dependence on pyrolysis temperature, reflecting the evolving carbon microstructure and interconnectivity of the particle skeleton. As shown in Figure 6, both modulus and strength respond nonlinearly to increasing temperature, while elongation at break reflects the material’s ductility and failure mode. At 800 °C, the carbon aerogel presents a relatively low modulus of 1489.56 MPa, but surprisingly, a moderate compressive strength of 46.84 MPa and the highest elongation at break (5.11%). This behavior suggests a highly disordered yet compliant amorphous framework that can deform to some extent before failure. The loose packing and disorder nature of the carbon, as confirmed by XRD and Raman, allow strain redistribution under load. At 1000 °C, the modulus slightly increases to 1525.11 MPa, but the strength drops to 31.12 MPa, and the elongation at break sharply decreases to 3.12%. After heat treatment at 1000 °C, the carbon skeleton begins to undergo partial densification, but the degree of carbon ordering is still insufficient, as reflected by the relatively high I_D_/I_G_ ratio in Raman spectra compared with higher-temperature samples. This intermediate state leads to inhomogeneous local graphitization and defect distribution, which weakens the structural integrity and makes the network more brittle and defect-prone, explaining the observed decrease in compressive strength relative to the 800 °C sample. A similar pattern is observed at 1200 °C, where although the modulus increases substantially to 2492.45 MPa, the strength only recovers to 60.47 MPa, and elongation remains low (3.13%). These samples exhibit higher stiffness but poor toughness.

CA-10%-1400 °C yields the best overall mechanical performance, with a modulus of 2934.59 MPa, a compressive strength of 110 MPa, and a moderate elongation of 4.21%. This balance of stiffness and ductility arises from the emergence of uniformly dispersed 2~3 nm graphitic nanocrystallines, which reinforce the amorphous matrix and enable enhanced stress transfer across the skeleton. The thickened particle necks formed during self-assembly remain intact after carbonization, further promoting load-bearing continuity. This hybrid nanostructure, consisting of rigid crystalline domains and an interconnected amorphous framework, provides both modulus enhancement and energy dissipation capacity, leading to exceptional mechanical synergy. Interestingly, at 1600 °C, although the modulus remains relatively high (2571.53 MPa), the strength decreases to 78.48 MPa, and elongation further drops to 3.84%. This decline is attributed to over-graphitization and crystallite coarsening, which induce localized stress concentration. The excessive rigidity of the highly graphitized skeleton reduces its ability to accommodate strain, resulting in reduced toughness and premature failure under compression.

Taking mechanical properties and characterization results together, it can be concluded that increasing temperature improves stiffness through enhanced carbon ordering, but only a moderate degree of nanocrystallization (1400 °C) yields optimal strength and ductility. This underscores the critical role of nanoscale crystalline reinforcement embedded within a well-connected network in achieving high-performance carbon aerogels. Excessive graphitization, while beneficial to thermal stability, can be detrimental to mechanical resilience. The graphitized nanocrystals formed during pyrolysis provided additional strength by enhancing interlayer stress transfer, while the neck structure of the particle packing ensured continuous load-bearing paths.

### 3.4. Effect of Closed-Cell Structure on Thermal Insulation Performance

**Figure 7:**
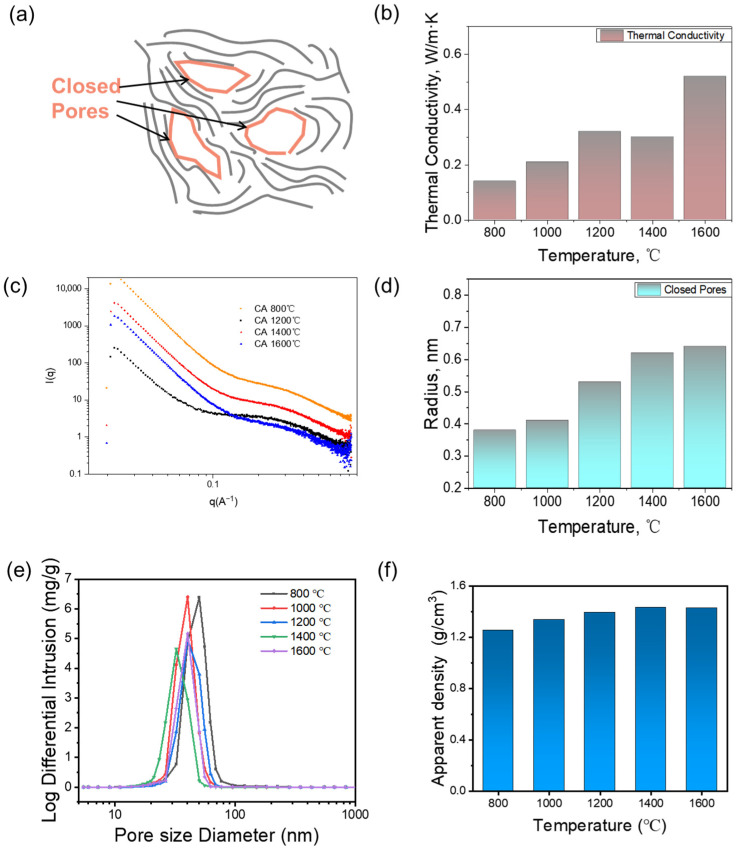
(**a**) Schematic illustration of closed pore structure in carbon aerogel, (**b**) thermal conductivity of CAs at different temperature, (**c**) SAXS curves of CAs at different temperature, (**d**) closed pore radius of CAs at different temperature, (**e**) open pore distribution of CAs at different temperature by Mercury Intrusion, and (**f**) skeleton density of CAs at different temperature.

The pyrolysis temperature significantly modulates the hierarchical structure of the carbon aerogels, thereby governing their thermal insulation properties. As shown in Figure 7a, the thermal conductivity increased markedly from 0.14 W·m^−1^·K^−1^ for CA-10%-800 to 0.21 W·m^−1^·K^−1^ for CA-10%-1000, and further to 0.32 W·m^−1^·K^−1^ for CA-10%-1200. Between 1200 °C and 1400 °C, the value remained relatively stable (0.30 W·m^−1^·K^−1^), while a significant jump to 0.52 W·m^−1^·K^−1^ occurred for CA-10%-1600. This trend is closely correlated with the evolution of closed pore size and skeleton characteristics. As shown in Figure 7c, SAXS curves exhibited a steep intensity drop in the low-q region and a pronounced shoulder at q range of 0.04–0.35 Å^−1^, evidencing a substantial population of nanometer-scale closed pores appeared. With increasing temperature, the shoulder peaks showed enhanced relative intensity and gradually shift to lower *q*, indicating pore volume and characteristic length of the porous network increased synergistically. To quantify structural differences among the CAs with different temperature, the curves were fitted with a Debye-type model [32],

(1) $$I\left(q\right)=\frac{A}{q^{4}}+\frac{B^{\prime }a_{1}^{4}}{{\left(1+a_{1}^{2}q^{2}\right)}^{2}}$$

where *A* accounts for Porod scattering from the external surface, $B^{\prime }$ describes the density–density correlation of the internal structure, $a_{1}$ is the correlation length, and D is the incoherent background. The radius of gyration of the nanopores, $R_{g}$, defined as the root-mean-square distance from the center of mass, is related to $a_{1}$ (Debye–Anderson–Brumberger form) by $R_{g}=\sqrt{3}\;a_{1}$. According to SAXS results (Figure 7d), the closed pore radius increased from 0.38 nm at 800 °C to 0.62 nm at 1400 °C, and slightly to 0.64 nm at 1600 °C, suggesting a gradual pore coarsening with temperature. For thermal transportation within the carbon aerogel backbone, the pore structure can be considered as defects for the heat transfer route. The higher the porosity, the more frequent the scattering of phonons at the pore interfaces, and the lower the thermal conductivity. Furthermore, micropores are also structures formed during the carbonization process. Micropores are more abundant in the temperature range of 800–1000 °C and gradually decrease as the temperature rises to 1200–1600 °C due to structural rearrangement and partial pore coalescence, while it generally has a negligible effect on thermal insulation [32,33].

The open pore structure also played a vital role for thermal insulation. Mercury Intrusion porosimetry was employed to analyze the open pore architecture and skeleton density of carbon aerogels subjected to various pyrolysis temperatures. As illustrated in Figure 7e, the pore size distribution revealed a relatively narrow peak in the meso–macroporous range (20–50 nm) across all samples. The nano open pores can suppress gas convection through the Knudsen effect, where gas molecules collide more frequently with the pore walls than with each other, effectively reducing the convective heat transfer. With increasing pyrolysis temperature, the mean pore diameter varied between 32 nm and 50 nm. This evolution suggests volume shrinkage during thermal treatment, accompanied by partial open pore collapse. Concurrently, the skeleton density increased from 1.25 g/cm^3^ to 1.43 g/cm^3^, indicating gradual densification and increased carbon framework connectivity through graphitization and loss of volatile components. At lower temperatures (800–1000 °C), the rise in thermal conductivity is primarily attributed to the beginning of graphitization and initial densification. From 1000 to 1400 °C, despite continued growth in closed pores and marginal increase in skeletal density, the conductivity plateaued. This plateau can be explained by the balance between enhanced phonon transport from structural ordering and phonon scattering due to residual turbostratic domains and pore interfaces. At 1600 °C, the thermal conductivity increased sharply. This can be ascribed to the formation of more continuous nanocrystalline graphite domains, which promote long-range phonon transport. Additionally, the enlarged closed pores and increased skeleton density reduce interfacial thermal resistance, further boosting heat conduction.

Taken together, the results reveal that thermal conductivity is governed by a synergistic interplay of closed pore structure, skeleton structure, and graphitic continuity. Moderate graphitization offers a balance between insulation and stability, while extensive ordering and densification at 1600 °C shift the material toward higher conductivity, potentially suitable for heat-spreading rather than insulating applications.

## 4. Conclusions

In this study, a molecular assembly strategy was developed to fabricate carbon aerogels by precursor of PF with polyurethane PUU through hydrogen-bond-assisted self-assembly and curing. This route enabled precise regulation of the precursor skeleton, where increasing PUU content led to controlled growth of primary particles (from ~109 nm to 1516 nm) and a transition from pearl-like to thick-necked architectures. Such preorganized frameworks served as a structural blueprint that was well-retained during carbonization. Pyrolysis temperature further modulated the hierarchical structure by inducing nanocrystallization, skeleton densification, and pore evolution. Mercury Intrusion and SAXS results revealed a dual structural transition, mesopore shrinkage, and backbone compaction, alongside coarsening of closed pores from 0.38 nm (800 °C) to 0.64 nm (1600 °C). The structure–property relationship exhibited a non-monotonic trend. Aerogels carbonized at 800–1000 °C maintained high porosity but lacked sufficient carbon ordering, resulting in low modulus and strength. At 1200 °C, enhanced stiffness emerged (modulus: 2492 MPa), but limited graphitization degree restricted mechanical reinforcement. The 1400 °C treated CA achieved an optimal balance: embedded nanocrystals (2~3 nm), continuous-necked particle connectivity, and preserved mesoporosity enabled superior performance with modulus of 2934 MPa, strength of 110 MPa, and thermal conductivity of 0.30 W·m^−1^·K^−1^. At 1600 °C, excessive graphitization improved skeletal density and thermal conduction (0.52 W·m^−1^·K^−1^), but reduced ductility and strength due to brittleness. The findings offer a generalizable framework for designing porous carbon materials with targeted performance for thermal protection and may expand the application boundaries of carbon aerogels in extreme environments.

## Data Availability

The original contributions presented in this study are included in the article/Supplementary Materials. Further inquiries can be directed to the corresponding authors.

## Supplementary Materials

The following supporting information can be downloaded at: https://www.mdpi.com/article/10.3390/nano15241874/s1, Figure S1. Schematic diagram of the covalent bonding between PUU (after end-capping with 3-aminophenol) and Phenolic Resin (PF). Figure S2. Dynamic light scattering analysis of PUU 10%, PUU 20% and PUU 30% PF-PUU in iso-propanol. Table S1. Table of carbonization temperature, density, and thermal conductivity for different samples. Table S2. Table of density and linear shrinkage for PF and samples with different PUU contents.

## Abbreviations

The following abbreviations are used in this manuscript:

CAs	Carbon aerogels
PUU	Polyurethane-urea oligomer
PF	Phenolic resin
SAXS	Small Angle X-ray Scattering
XRD	X-ray diffraction
