# Supplementary material for: Tailoring Architecture of Carbon Aerogel via Self-Assembly Template for Balanced Mechanical and Thermal Insulation Performance
Source: Nanomaterials (Basel). 2025 Dec 13;15(24):1874. doi: 10.3390/nano15241874 (PMC12735542; doi:10.3390/nano15241874)
Supplement: Supplementary file 1 [file nanomaterials-15-01874-s001.zip › nanomaterials-4003263-supplementary.pdf]

Supporting Information for

Tailoring Architecture of Carbon Aerogel via Self Assembly Template for Balanced Mechanical and Thermal Insulation Performance

Lei Yang [1, 2, #], Xianxin Shao [3, #], Lin Lu [2], Xiaoyan Chen [2], Yiming Yang [3, *], Hao Li [3], Yiqiang Hong [2] and Yingjie Qiao [1, *]

1 College of Material Science and Chemical Engineering, Harbin Engineering University, Harbin 150001, China

2 Beijing System Design Institute of Electro-Mechanic Engineering, Beijing 100854, China;

3 Key Laboratory of Science and Technology on High-tech Polymer Materials, Institute of Chemistry Chinese Academy of Science, Beijing 100190, China; shaoxianxin@iccas.ac.cn

* Correspondence: qiaoyingjie@hrbeu.edu.cn (Y.Q); yiming924@iccas.ac.cn (Y.Y)

# These Authors Contributes Equally

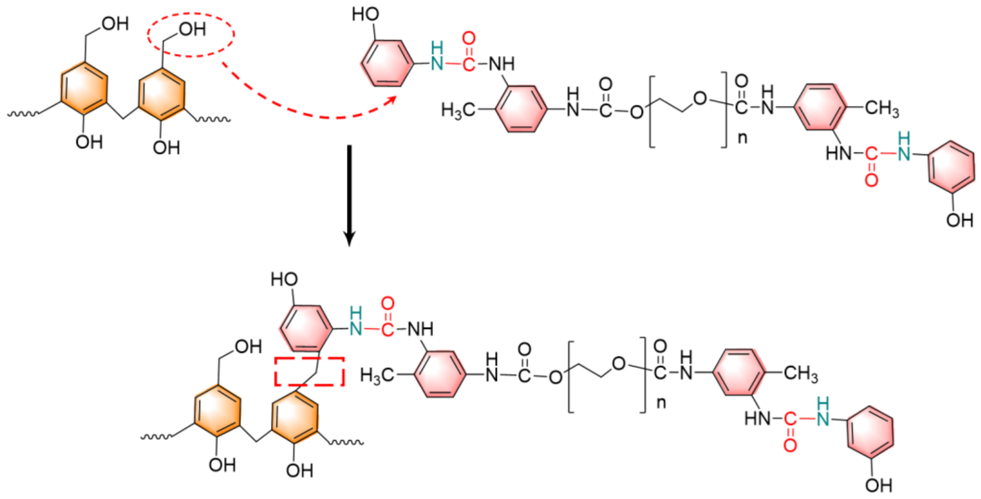

Figure S1. Schematic diagram of the covalent bonding between PUU (after end-capping with 3-aminophenol) and Phenolic Resin (PF).

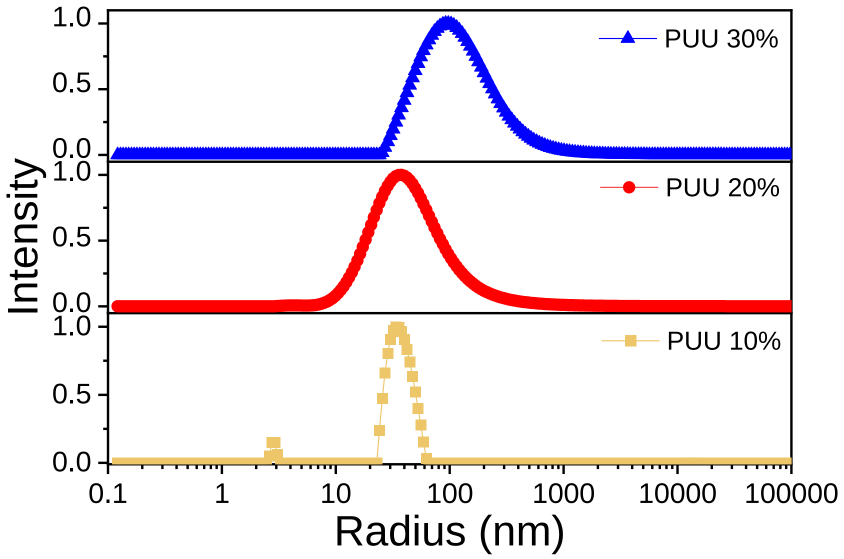

Figure S2. Dynamic light scattering analysis of PUU 10%, PUU 20% and PUU 30% PF-PUU in iso-propanol.

Table S1. Table of carbonization temperature, density, and thermal conductivity for different samples

Temperature,℃	Density, g/cm3	Thermal conductivity, $W \cdot m^{-1} \cdot K^{-1}$
800	0.544	0.14
1000	0.553	0.21
1200	0.563	0.32
1400	0.554	0.30
1600	0.589	0.52

Table S2. Table of density and linear shrinkage for PF and samples with different PUU contents

Sample	Density, g/cm3	Linear Shrinkage Rate, %
PF	0.39	7.6
PUU5%	0.36	3.2
PUU10%	0.35	1.1
PUU20%	0.35	1.1
PUU30%	0.34	0.6
